# How to talk so kids will listen & listen so kids will talk: a randomized controlled trial evaluating the efficacy of the how-to parenting program on children’s mental health compared to a wait-list control group

**DOI:** 10.1186/s12887-018-1227-3

**Published:** 2018-08-02

**Authors:** Mireille Joussemet, Geneviève A. Mageau, Marie-Pier Larose, Mélanie Briand, Frank Vitaro

**Affiliations:** 1 Psychology Department, University of Montreal, Montreal, Canada; 2 Department of Social and Preventive Medicine, University of Montreal, Montreal, Canada; 3 School of Psycho-education, University of Montreal, Montreal, Canada

**Keywords:** Health promotion, Preventive psychiatry, Child mental health, Parenting program, Parent-child relations, Optimal parenting style, Autonomy support, How-to parenting program

## Abstract

**Background:**

Basic parenting research reveals that child mental health is associated with optimal parenting, which is composed of three key dimensions (structure, affiliation and autonomy support). The present study aims to test the efficacy of the parenting program “How to talk so kids will listen & listen so kids will talk” (French version), thought to address all of these dimensions, in promoting children’s mental health. We predict that the How-to Parenting Program will promote child mental health by fostering optimal parenting.

**Methods:**

In this randomized controlled trial (RCT), the seven-week parenting group was offered to parents of 5- to 12-year-old children, in their local grade school. Children’s mental health assessments were questionnaire-based (parent, child and teacher reports) and took place at pre- (T1) and post- (T2) intervention as well as at 6-month (T3) and 1-year (T4) follow-ups. We compared children whose parents took part in the program with children whose parents did not take part in it until the completion of the trial (i.e., 1 year wait-list control groups). The primary outcome is children’s psychological problems (externalizing and internalizing). Secondary outcomes include parenting, the putative mediator of the expected benefits of the program on child mental health, as well as positive indicators of child mental health (strengths and subjective well-being) and parents’ own mental health.

**Discussion:**

To our knowledge, this is the first RCT to test the efficacy of the “How to talk so kids will listen & listen so kids will talk” program in promoting child mental health. In addition to the close correspondence between basic parenting research and the selected program, strengths of this study include its feasibility, monitoring of potentially confounding variables, ecological validity and inclusion of positive indicators of mental health.

**Trial registration:**

Current clinical trial number is NCT03030352. Ongoing study, retrospectively registered on January 2017. No amendment to initial protocol.

**Electronic supplementary material:**

The online version of this article (10.1186/s12887-018-1227-3) contains supplementary material, which is available to authorized users.

## Background

Parents are not only the individuals who care the most about their children’s development and well-being, they are also a main determinant of these outcomes. Indeed, among environmental factors, parenting quality is the most widely accepted predictor of children’s mental health [1]. The goal of the present study is to promote children’s mental health by fostering optimal parenting.

Mental health can be defined as the absence of psychological problems and the presence of strengths and well-being [2–4]. First, there are two broad categories of psychological problems during childhood: externalizing (E) and internalizing (I) problems [5, 6]. Children with E problems (e.g., opposition, aggression) display *under*controlled behaviors [7, 8], lack self-regulation, and direct their negative emotions against others [7]. In contrast, children with I problems (e.g., anxiety, depression) display *over*controlled behaviors [6, 9, 10], have overly rigid self-regulation, and direct their negative emotions toward themselves [11]. Developing self-regulation (absence of E problems) that is devoid of rigidity and ill-being (absence of I problems) is thus at the root of child mental health. Second, in order to gain a complete account of children’s mental health, it is essential to consider children’s socio-emotional strengths and well-being in addition to their psychological problems [3, 12, 13]. Examples of positive indicators of mental health include positive indicators of emotional and behavioral self-regulation (e.g., frustration tolerance, prosocial behaviors) as well as indicators of subjective well-being (e.g., life satisfaction, social skills).

Given the vast influence that parents have on their children’s mental health [1, 14], parenting training has been proposed as an effective way to prevent and even treat child psychopathology [15, 16]. The vast majority of parenting programs target families in which children already display problems (mostly E) or are at risk of doing so (i.e., indicated or selected prevention strategies) and focus on children’s behaviors [17]. There has been relatively less attention paid to the promotion of child mental health (universal prevention strategy). The present study offers the opportunity for parents of the general population to improve their parenting style and, in turn, promote their children’s mental health. Parenting programs targeting the parenting style (rather than children’s behaviors) should be best-suited for the universal promotion of child mental health.

Research in developmental psychology has shown that optimal parenting is composed of three key dimensions, namely structure, affiliation and autonomy support [18, 19]. Affiliation, the opposite of hostility and rejection, refers to warmth, care and acceptance [20, 21]. Structure, the opposite of permissiveness, refers to clear and consistent expectations and consequences [22, 23]. Finally, autonomy support (AS) is the opposite of controlling parenting [18, 24]. It refers to consideration and respect for children’s own ideas, feelings, and initiatives [25, 26]. When making requests, AS has been operationally defined as the provision of empathy, rationale, choice and non-controlling language [27].

Parental AS is a powerful determinant of children’s mental health and well-being [28]. AS predicts a host of positive child outcomes even after accounting for the effects of other positive parenting dimensions [29–31]. Importantly, a meta-analysis [32] reveals that empathy (e.g., empathic listening, following child’s interest), a key component of AS, is one of the most active ingredients in successful parenting training, along with positive interactions and consistent responding. In sum, motivation and parenting research suggests that AS, along with affiliation and structure, should be an integral part of any parenting program aiming to improve parenting. The goal of the present study is to assess the impact of a parenting program that adopts this broader scope and teaches all three dimensions of the optimal parenting style on children’s mental health.

While most parenting programs target structure and affiliation [15, 33–35], one program in particular, “How to talk so kids will listen & listen so kids will talk” [36] (called the How-to Parenting Program herein) truly addresses all three key dimensions of optimal parenting by incorporating AS practices in a vast array of daily situations. This program stems from parenting groups led by the child psychologist Haim Ginott (1922–1973) whose writings [37–39] inspired the operational definition of AS (Koestner et al., 1984). We thus assessed the efficacy of this parenting program because it addresses all three key dimensions of optimal parenting and truly captures the essence of AS.

Faber and Mazlish wrote the “How to talk so kids will listen & listen so kids will talk” book [40] in 1980 to help other parents by sharing the knowledge they had gained by taking part in parenting groups led by Ginott. Originally written in English, this book has been translated in more than 20 languages and remains a best-selling parenting book. The wide dissemination of this program represents another reason for its assessment, which we considered a social and ethical imperative. We thus formed partnerships with local grade schools to implement and assess the How-to Parenting Program.

### How-to parenting program

#### Overview

“How to talk so kids will listen & listen so kids will talk” is a book [40] and a group workshop [36]. The latter is available in an audio (CD) and video (DVD) format. Through these recordings, its authors present the parenting skills to participants and provide specific instructions as to when to play the CD/DVD, pause it, complete exercises and have open discussions. The audio and video formats are designed to allow any group of parents to receive training since the designated leader does not need training or certification.

#### Content

The program’s main themes and skills are summarized in Table 1, along with examples. In our view, the three key dimensions of the optimal parenting style are addressed through these skills, which are depicted in common daily family situations. First, many of the How-to Parenting Program’s skills utilize AS. Empathy, a key component of AS, is foundational in this program (chapter 1). In addition, parents learn how to encourage and support children’s initiatives and agentic functioning (chapter 4) and how to avoid confining them in certain roles (chapter 6). This open, informational, considerate and flexible style perfectly matches the definition of parental AS and allows it manifestation in a wide range of daily situations.

**Table 1:** Skills Taught in the How-to Parenting Program

Session/Chapter title		Skills	Examples
Session 1/ Chapter 1	Helping children deal with their feelings	- Listen to him/her with full attention;	Look at the child when s/he speaks.
		- Acknowledge with a word, and/or a sound;	“Oh…”; “Hm”
		- Try to name the child’s feeling;	“That can feel scary”
		- Give him/her what s/he desires in fantasy.	“I wish I could make a snack appear for you right now”
Session 2/ Chapter 2	Engaging cooperation	- Describe what the problem is;	“There are boots in the middle of the hallway”
		- Provide some more information;	“It’s hard to walk when boots are blocking the way and wetting the floor”
		- Remind the child with just one word;	“Kids, the boots”
		- Express your own feelings without attacking the child’s character;	“I feel irritated when I come back home and can’t walk in the hallway”
		- Write a note.	“Please bring us back on our rack” (*signed: your boots)*
Session 3/ Chapter 3	Alternatives to punishments	- Express own feelings without attacking the child’s character;	“I don’t like to see food residues on the couch”
		- State your expectation;	“I expect eating to take place in the kitchen”
		- Show him/her how to make amends;	“This couch needs to be cleaned. Here’s a wet sponge with some soap on it”
		- Give him/her two options;	“You can either eat your snack in the kitchen before watching TV or watch TV without a snack”
		- Take action if needed;	After giving options (see above), take away the snack.
		- Problem-solve with child.	Acknowledge child’s feelings; Express yours; Brainstorm (write child’s ideas and your own); Select one idea, Plan and implement it.
Session 4/ Chapter 4	Encouraging autonomy	- Let him/her decide;	“Do you want the blue or the red shirt?”
		- Respect the child’s struggle;	“Pouring milk in a glass can be tricky, sometimes it helps to use a wide glass”
		- Limit the number of your questions;	Let child talk about his/her day when s/he wants to.
		- Don’t rush to answer his/her questions;	“Interesting, why do *you* think kids lose their teeth?”
		- Promote some outside resources;	“I wonder what the dentist would say”
		- Don’t take away the child’s hope.	“An astronaut! What an interesting career.”
Session 5/ Chapter 5	Descriptive praise	- Describe the child’s behavior or accomplishment;	“I see toys on their shelf”
		- Describe own feelings;	“It feels good to sit on the couch easily”
		- Summarize the child’s behavior with a noun.	“That’s what I call *organization*”
Session 6/ Chapter 6	Freeing children from playing roles		Example: the “sore loser”
		- Notice counter role behavior from the child;	“You shook the winner’s hand”
		- Provide him/her with counter role opportunities;	“Let’s play a game of …”
		- Let the child overhear positive comments;	“Suzie congratulated me when…”
		- Model appropriate behavior;	“Congratulations for winning this game!”
		- Recall one of the child’s counter role behavior in the past;	“I remember when you congratulated me for winning at …”
		- If s/he reverts to an old role, state your feeling and expectation.	“I expect you to congratulate the winner after a match”
Session 7	Integration	Open, guided discussion; Activity about managing typical parent-child interactions by integrating various skills; Description of participants’ accomplishments in learning skills.	

Second, the How-to Parenting Program addresses structure and teaches parents how to provide it. A key distinction is made between children’s emotions and behaviors, by stating that whereas all feelings can be accepted, not all behaviors should be [39]. Parents learn how to communicate their expectations (chapter 2), follow through with logical consequences (e.g., make amends; chapter 3), use problem-solving for recurrent problems (chapter 3) and give feedback (chapter 4). These skills are coherent with the provision of clear and consistent rules, expectations and consequences inherent in the dimension of parental structure [22] and help parents guide and limit their children’s behaviors.

Third, affiliation is pervasive in the How-to Parenting Program (chapters 1 to 6), as creating and maintaining a positive parent-child relationship is at the heart of this program. Rather than using incentives, or contingent attention/regard, this material targets the way parents communicate with their children, which, in turn, can strengthen (vs. erode) the relationship. Parents thus learn skills that help them listen more empathically and respond to their children in a way that conveys their unconditional acceptance. With its broad scope and its concrete skills, the How-to Parenting Program should be beneficial by promoting a parenting style shown to foster children’s mental health.

### Pilot study

As a preliminary step in testing this hypothesis, we conducted a pilot study using a pre-post intervention design. After gaining permission from Faber and Mazlish (personal communication, September 2007), we offered the How-to Parenting Program to 11 groups of parents (*N* = 93) in their grade schools [41]. Attendance was high, as 85% of parents attended six to eight of the eight sessions offered. Most parents completed their questionnaires even though there was no compensation in this study. Attrition rates were of 10, 39 and 48% at post-intervention, 6-month and 1-year follow-ups, respectively. When two independent coders evaluated audiotaped material, the average content fidelity score was 85%, with an inter-rater reliability of 91% [42].

Results of this pilot study showed that after having taken part in the How-to Parenting Program, parents provided more structure, AS and affiliation than before [41]. We also found that children’s mental health at post-intervention was better than at pre-intervention and that these improvements, moderate to large in size, were still present 6 and 12 months later [43].

Although these results are promising, the lack of a comparison group did not allow for an adequate control of the impact of the passage of time on the outcome variables. We thus designed a randomized controlled trial (RCT) with a wait-list control group. We favored a wait-list control group to assess the absolute (vs. relative) efficacy of How-to Parenting Program because this type of control group should facilitate the comparison of the effects of the How-to program to the ones obtained for other programs (using Cohen’s d) on comparable measures. Indeed, most (83.1%) parenting programs are evaluated using a wait-list control group [32].

## The Present Study

### Objectives

The aim of the present study was thus to test the efficacy of the How-to Parenting Program (French version) on children’s mental health. Specifically, we aimed to assess whether this parenting program would not only foster decreases in children’s E and I problems but also increases in children’s strengths and subjective well-being.

#### Design

To reach these objectives, we used a prospective, superiority RCT with two parallel arms comparing children whose parents took part in the French How-to Parenting Program with children whose parents did not take part in the parenting program until the completion of the trial (i.e., one year wait-list control groups). We planned to use four waves of recruitment. Questionnaire-based assessments took place at pre- (T1) and post- (T2) intervention as well as at 6-month (T3) and 1-year (T4) follow-ups.

#### Primary hypothesis

We expected that children of parents in experimental groups would experience fewer parent-reported I and E psychological problems over time whereas children of parents on the wait-list would not show improvements during that year (i.e., stable or increasing psychological problems).

#### Secondary hypotheses

We also expected that children of parents assigned to experimental groups would experience decreases in teacher-rated E and I problems as well as increases in teacher-rated strengths and in child-reported well-being over time, whereas children whose parents were on the wait-list would not show such improvements during that year (i.e., stable or increasing psychological problems; stable or decreasing strengths and well-being).

We also expected that parents assigned to experimental groups would show improvements in parenting over time (i.e., increases in parental affiliation, structure and AS), whereas parents in control groups would not show such improvements (i.e., stable or declining parenting quality) during that year. Lastly, parents assigned to experimental groups were expected to experience improvements in their own mental health (decreases in symptoms and increases in well-being) compared to parents in control groups who would not show such improvements over time (i.e., stable or declining parental mental health). Based on parenting and motivation research, we also expected that increases in parental affiliation, structure and AS would mediate the expected improvements in children’s mental health.

## Methods

### Participants

The present study took place in public grade schools in the greater Montreal area, in the province of Quebec (Canada). Adopting a universal approach, the How-to Parenting Program was offered to all parents of recruited grade schools.

Assessments were made by participating parents, their participating child and the child’s teachers. Teacher reports were collected to test the generalization of the program’s impact (children’s improved mental health at school) and to gather reports from blind participants, thereby reducing social desirability attached to parent reports. Inclusion criteria for parents were: having at least one child attending a participating grade school, aged between 5 and 12 years old. Inclusion criteria for teachers were: currently teaching a child whose parent participates in the study and who consented to their targeted child’s teacher’s participation. Inclusion criteria for children were: being 8 years or older and having parental consent. Exclusion criteria for all were: inability to communicate in French.

For parents who had more than one child attending grade school, we guided them in identifying their “targeted” (i.e., participating) child. To avoid any bias that could be introduced by letting parents choose the targeted child themselves, we asked parents to select the child who was 8 years or older. If parents had more than one child over 8 years old, we asked them to select their child closest to 9 years of age. Similarly, if parents had more than one child under 8 years old, they were also asked to select the child closest to 9 years of age.

### Intervention

#### How-to parenting Program’s general format

Seven weekly sessions took place at children’s grade schools, from 7 to 9:30 p.m. The French version of the “How to talk so kids will listen & listen so kids will talk” workshop was offered by two trained group facilitators (this version is manualized; verbatim is based on the English audio format [44]). The workshop closely matches the book: the first six sessions cover the first six chapters and the last session is a general, integrative overview. Parents can learn an average of five skills per week during the six topical sessions. A common feature throughout the various communication skills is the use of an informational (vs. evaluative) style that doesn’t target the child’s character. Indeed, whether praise is given or a problem is described, parents are invited to focus on the task (e.g., “I see books back on their shelves and toys in their box!”), refraining from alluding to the child’s character or worth (e.g., “You are such a good girl”).

During the first session, the “rules of conduct” (e.g., confidentiality) are presented, and parents are invited to introduce themselves briefly by talking about what surprised them in their parenting role. This introduction is meant to address parents’ motivation by eliciting their wishes and expectations, which in turn predicts successful behavioral change.

Through sessions 1 to 6, a total of 30 skills are taught. Every session begins with a discussion about the previous week’s homework (except in session 1). Facilitators devote up to 30 min to welcome and listen to parents’ account of their new skill implementation, whether it seemed successful or not. Next, the main theme is introduced with a perspective-taking exercise. Parents are first placed in “children’s shoes” by listening to typical comments/requests that children often hear and they are then encouraged to describe how they feel. The presentation of alternative communication skills follows, illustrated in comic strips. The rest of the session is composed of various exercises, allowing parents to practice each skill. Most exercises are role-playing activities, often conducted in dyads. Other exercises take place in subgroups and still others are conducted individually. Each involves note-taking in a workbook. In general, parents describe how they feel in a scenario, when playing the role of a given child or a given parent. Group members then share their experiences and a structured discussion addresses participants’ reactions and questions. Before leaving, the homework is introduced by facilitators who stress the importance of giving skills a try at home, with their own child or children.

The 7th session is a structured discussion to review, discuss and integrate the recently learned parenting skills. During that overview, parents think of a challenging situation with their child and all participants are invited to suggest how their newly acquired skills could be useful. At the end, facilitators give each participant a colored summary sheet as well as a stack of small illustrated cards that summarizes all skills (created for the present RCT). They also acknowledge parents’ efforts and accomplishments in their discovery and early mastery of many new skills and stress the importance of cultivating a patient, compassionate attitude toward themselves.

#### Material

All parents had their own workbook to complete exercises during the program’s sessions and for their weekly homework. They also had a copy of the book [45] to complete the assigned readings. The participant workbook was provided free of charge to parents, but parents were asked to make a 25$ (Canadian; CAN) deposit for the book. This amount was given back at the end of the program, unless parents wished to keep their book. We lent the book without deposit to parents who expressed that this expense was difficult for them.

### Adherence

A large number of facilitators received training, as a large pool was needed for this study (up to eight leaders available per condition, per year). In line with the inclusive stance adopted by the program’s authors, there was no “required qualification” to become a facilitator. Some interested facilitators were graduate students in psychology, others were parents and/or adults involved in education or a related domain.

#### Facilitators’ training

Given that with the French version, group facilitators could not rely on audio or video recordings, they received a 3-day training to promote adherence. This training was provided by a mental health professional who has had a long experience offering the How-to Parenting Program. In addition to being exposed to the program’s content, facilitators also learned about the process of facilitating it. Topics included avoiding acting as an “expert” and using the program’s communication skills oneself when facilitating the program. Facilitators were also encouraged to convey unconditional regard, be empathic and foster self-compassion. Finally, this training also addressed some of the particularities associated with facilitating a group within a RCT, such as content fidelity. These included having facilitators’ own voice recorded during all sessions, following the workshop material as much as possible, and refraining from integrating ideas, exercises, or opinions from other sources.

Supervision meetings took place before and after each wave, during which facilitators were offered and shared a wide range of useful information, which was written in a “facilitator’s guide”, updated yearly. This dynamic guide comprised both practical (e.g., material provided) and process-oriented guidelines (e.g., avoid trying to convince a parent appearing skeptical). Each team was composed of a more and a less experienced facilitator, who shared their experience and questions after each session. Individual supervision was also available if needed, offered by one of the principal investigators, also a licensed psychologist.

#### Adherence monitoring

The five aspects of program integrity were assessed, as it is essential to evaluate whether the intervention was offered completely (content fidelity) and adequately (process fidelity [46]). After each session, facilitators were asked to rate the percentage of material that was covered using a brief weekly online questionnaire. In addition, all sessions were audiotaped to permit two independent coders to verify content fidelity [46, 47]. Specifically, they assessed whether each activity was completed or not using a checklist [42, 46]. At the end of their 7-session program, facilitators rated each parent’s general involvement and enthusiasm to measure their responsiveness [47]. Process fidelity was assessed by parents, who rated their group facilitators’ empathy, enthusiasm and preparedness [46].

#### Exposure

To assess participants’ exposure to the program, group facilitators took attendance on-site, using a list of participants. They then transcribed this information when they received their brief post-session questionnaire, online.

#### Differentiation

While some participants in the experimental condition may have not fully engaged in the program, some parents in the wait-list condition might have gained access to some of the program’s content. Indeed, contamination was possible since we decided to randomly assign participants within each participating school. This procedure was chosen over a cluster randomized trial to avoid conflating our experimental manipulation with schools’ characteristics, such as their size, socio-economic status (SES), educational philosophy, and to remove this unexplained between-school variability from the between-group main effect, thereby increasing statistical power.

Because all parents in the study heard about the program during the information meeting (see Recruitment section below), some parents assigned to the wait-list condition may have decided to buy and read the book (and try some skills). However they could not take part in the program, which presumably fosters increased learning due to group participation (e.g., weekly sessions with facilitators and other parents, exercises, homework, discussion and modeling). To control for this potential confound, we asked parents in the wait-list condition whether they had bought and read the book. Finally, to ensure differentiation between the How-to Parenting Program and other interventions as well as between the experimental and the control conditions, parents in both conditions were asked to document any intervention and/or a therapeutic activity used for their family.

### Outcomes

Most parenting program studies emphasize E problems since they are disruptive, but I problems are also an important source of suffering and need to be prevented. Moreover, assessing the level of children’s strengths and well-being allows for a complete account of their mental health. The primary outcome measure is children’s I and E mental health problems, as assessed by their parents. Secondary outcome measures include other indicators of children’s mental health, as teachers rated children’s E and I problems and socio-emotional strengths and children rated their subjective well-being. Secondary outcomes also include the three dimensions of optimal parenting (as perceived by children, in addition to parental reports) and parents’ own mental health (indicators of both symptoms and well-being).

### Participant timeline

All parents and children completed their questionnaires before randomization (pre-intervention; T1), 1 week after the seven-week program (post-intervention; T2), and again at 6-month (T3) and 1-year (T4) follow-ups to assess change over time (see Table 2). Teachers each completed two questionnaires, since children’s teachers at the beginning of the study (T1 and T2, in February and April) were not the same teachers as during the last part of the study (T3 and T4, in October and April of the following school year).

**Table 2:**
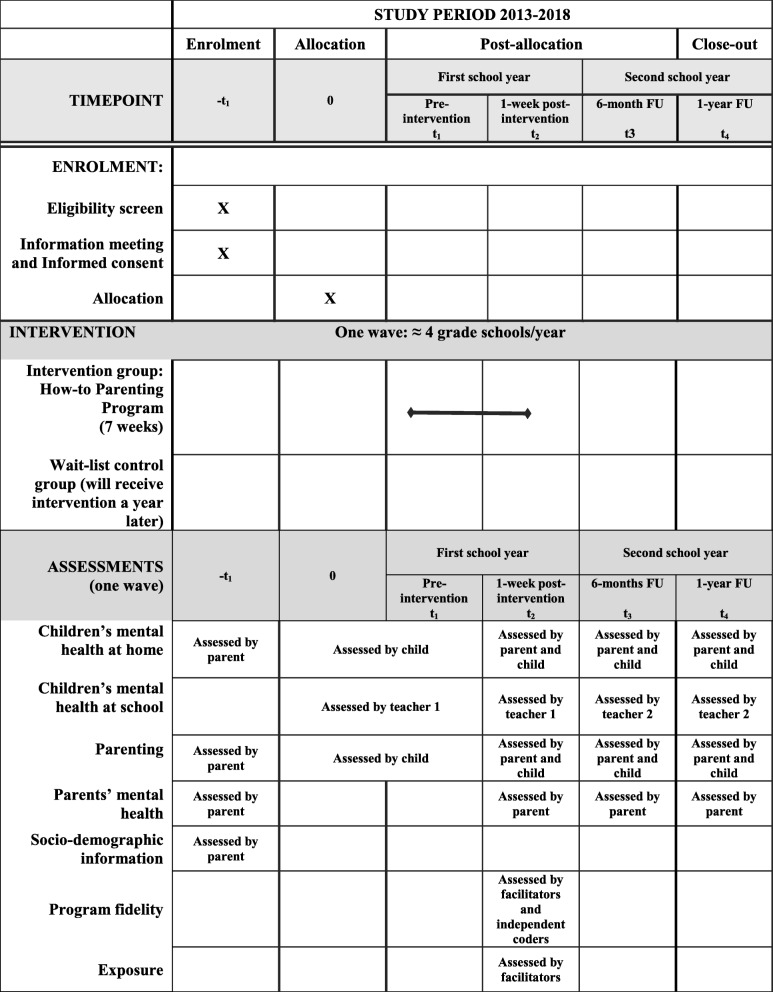
Schedule of Enrolment, Intervention and Assessments of the How-to Parenting Program RCT

### Sample size

The pilot study suggests that medium effects can be expected (i.e., Cohen’s d around.5) [48] and the primary focus of the present study pertains to cross-level interactions. Hox [49] suggests that these effects depend more strongly on the number of groups than on the total sample size. Tabachnick and Fidell [50] further suggest that sufficient power for cross-level effects is obtained when the number of groups is 20 or larger, whereas Paterson & Goldstein [51] recommend having at least 25 groups. Following these recommendations, our goal was to have 32 level-3 units (parenting groups), 256 level-2 units (parents), and 1024 level-1 units (time points). Sufficient power was expected since this number of parenting groups is above the recommended threshold and allows for recruitment drawbacks.

### Recruitment

**Fig. 1:**
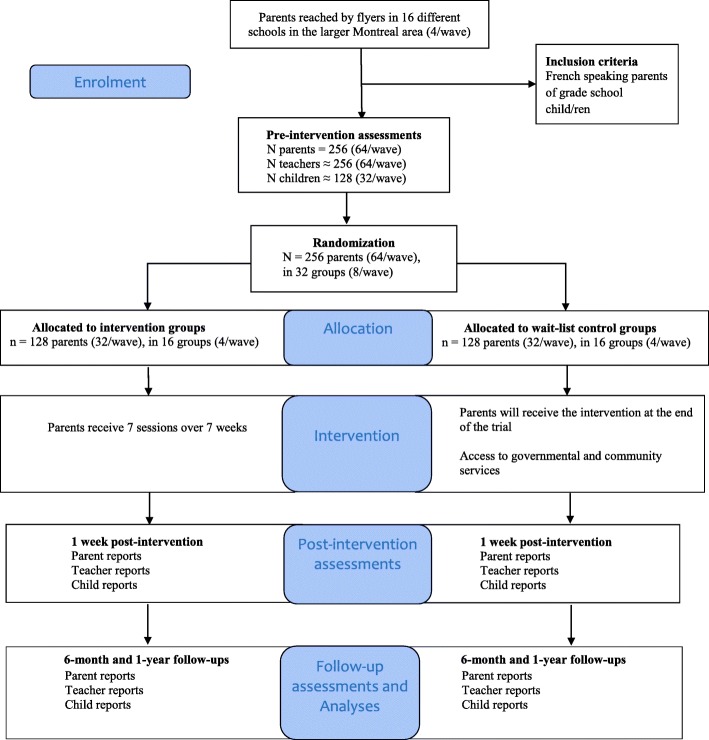
Flow of participants for the How-to Parenting Program RCT, planned over four waves

The goal was to conduct the study within four grade schools per year, for 4 waves (recruiting about 64 parents to form 8 groups at each wave; see Flowchart in Fig. 1). The RCT began after obtaining ethics approval and funding. We first sought approval from three school boards, a prerequisite for soliciting school principals. We then sent information to school principals by mail at the beginning of each wave (September), who could contact the research coordinator for further information if they were interested in this program implementation and evaluation. There was no inclusion or exclusion criterion for school recruitment; all schools were invited. Given that we did not target specific types of schools nor SES neighborhoods, school participation first depended on school principals’ interest. When a school principal was interested in our study, information was given to all families by sending an information flyer via children’s schoolbag, in December. Parents then communicated their interest in the program by returning the flyer’s response section (reply slip). We asked teachers to refrain from recommending the parenting program to certain parents, to highlight its universal and voluntary nature. Recruitment thus also depended on each particular school’s general level of parental interest since the next recruitment stage (information meeting) could take place solely in schools in which a large number of parents returned their reply slip.

If both parents of a same family expressed their wish to take part in the parenting program, we allowed them to do so (when there was enough space in a group) although data from the second, “duplicate” parent would not be used in statistical analyses. Identifying which parent was the participating one was decided by randomly choosing one of their sealed envelopes. Whether one or two parents participated in the program was coded, to examine whether this factor influences the program’s efficacy.

### Consent and allocation

Information meetings for parents were held in schools in January. One of the principal investigators met with interested parents to provide them with information about the parenting program and its assessment. Parents were thus informed about the How-to Parenting Program, the random assignment, their voluntary participation as well as that of their participating child and his/her teachers. Parental consent forms were filled out at the end of that information meeting. This consent form comprises three distinct sections, allowing parents to give their consent (or not) separately for (a) their child’s participation, (b) their child’s teachers’ participation and (c) their own.

Random assignment of families was made after parents’ T1 questionnaires were collected, within each school. The research coordinator extensively shuffled the sealed anonymous envelopes containing T1 questionnaires before randomly assigning them in one of the two conditions. Next, parents received a phone call informing them of the group they were assigned to (either in the group beginning the following spring or in the group beginning during spring of the following school year; see Fig. 1 and Table 2).

#### Blinding

Since parents knew in which condition they were assigned, their children may also have been aware of it. However, all research assistants (RAs) collecting child reports were blind to the intervention conditions, according to PROBE methodology to reduce assessment bias. Moreover, all teachers were asked to refrain from trying to know if a given pupil’s parent was taking part in the parenting program.

### Data collection methods

#### Parents

At the end of each information meeting, parents who had decided to take part in the study filled out a T1 paper-pencil questionnaire on site (see Instruments section below), after completing their consent form. When filling-out their T1 questionnaire, parents indicated whether they preferred to receive paper-pencil or online questionnaire. We thus either sent a paper-pencil version of T2, T3 and T4 by mail or provided parents a link, via email.

We collected all parent-reports (PR) at T1, prior to randomization. We also aimed to collect all of the T1 child-reports (CR) and T1 teacher-reports (TR) before the first session of experimental groups. We coded whether any of the CR or TR were collected after that, to verify whether including them influences the obtained results.

#### Teachers

The research coordinator met with participating schools’ teachers during one of their scheduled meetings, to briefly provide them with key information about the study. Teachers learned about the overall procedure and about what their possible participation would entail, i.e., filling-out a questionnaire about one or more of their pupils, on two occasions (either at T1 and T2, or at T3 and T4). Since children move to the next grade the following year, new teachers were also contacted and asked to fill-out the third (T3) and last (T4) TRs. All teachers for whom parental consent were obtained received their own consent form and paper-pencil questionnaires, in their school mailbox.

#### Children

Within each school, a RA met with participating children (individually or in small groups of a maximum of four children) in an available quiet room (e.g., school library) during a time that did not include any test or special activity. The RAs first informed children that their parent had agreed to participate in a study, without mentioning the parent’s participation to the parenting program. They then invited the children to fill out a questionnaire but specified that even though their parent gave them permission to participate, they could decide for themselves if they wished to participate or not. All children thus gave their verbal assent for their participation*.* Children completed paper-pencil questionnaires on their own but the RA remained available to answer questions about the questionnaire and study, if needed.

#### Group facilitators

Group facilitators audiotaped the sessions, answered a short questionnaire at the end of each session about the material covered, and monitored parents’ attendance. We also collected information about facilitators’ experience (in years), their age and sex, and whether they had children of their own to control for these factors, if needed. Each facilitator signed an informed consent form before providing this information.

### Distinguishing the program’s implementation from its evaluation

To reduce assessment bias, we ensured that parents made a clear distinction between the parenting program (which we called “the workshop”) and its evaluation (which we called “the study”). Second, we explained that compensation was contingent upon questionnaire completion, not on program participation. We also made this distinction salient by assigning different tasks to different members of our team. The research coordinator and RAs (rather than facilitators) took care of all research communication and procedures (i.e., questionnaires, consent forms, compensation) to foster role clarity. Group facilitators were asked to avoid talking about the study and to refer parents to the research coordinator if questions about the study arose.

### Instruments

#### Primary outcome

At each assessment time (pre-intervention, 1-week post-intervention, 6-month and 1-year follow-up), child’s mental health was assessed with different questionnaires via three different assessors (i.e., children themselves, their parent and their teachers). First, parents were asked to evaluate their child’s mental health using the two subscales - I and E problems - of the *Child Behavior Checklist* [52] (CBCL), a common outcome in trials assessing parenting programs. The CBCL is one of the most widely used validated instruments to assess children’s mental health. The E syndrome (Cronbach alphas T1/T2 = .88/.85 in our pilot study) reflects rule-breaking behavior and aggressive behavior whereas the I syndrome (Cronbach alphas T1/T2 = .81/.78 in our pilot study) reflects problems of anxiety/depression, withdrawal/depression and somatic complaints.

#### Secondary outcomes

##### Complementary measures of child mental health

Children were asked to evaluate their own well-being using measures of positive affect, life satisfaction and self-esteem. Children’s positive affect scale was assessed with an adapted scale [53] based on the *Positive and Negative Affect Schedule* (*PANAS) for children* [54, 55] and used in our pilot study [41]. This subscale includes ten positive emotion items, chosen for their simplicity. This French subscale showed good internal consistency in our pilot study (Cronbach alphas T1/T2 = .86/.88). Children’s self-esteem was measured with the *Rosenberg’s Self-Esteem Scale* [56, 57], one of the most widely-used measures to assess children’s global self-esteem. It assesses the extent to which children have a positive attitude toward themselves, and shows good construct and convergent validity [57] as well as good internal consistency (Cronbach alphas T1/T2 = .71/.83 in our pilot study). Finally, items about children’s life satisfaction were drawn from the French version of the *Satisfaction with Life Scale* [58, 59], a subscale which demonstrated good internal consistency (Cronbach alphas T1/T2 = .89/.93) in our pilot study.

Children’s mental health problems and socio-emotional strengths were also assessed by their current school teacher. Teachers were asked to fill-out the *Teacher-Child Rating Scale* [60] (TCRS). The problem subscales of the TCRS assess I (shy-anxious) and E (acting-out) problems whereas the socio-emotional subscales tap frustration tolerance, task orientation and social skills, important self-regulatory skills.

##### Parenting

The three dimensions of an optimal parenting style (structure, affiliation and AS) were assessed at each assessment time, by both parents and children, using scales drawn from well-validated parenting questionnaires, translated to French using back translation and adapted for children when needed.

Parents completed the Laxness subscale of the *Parenting Scale* [61] to rate how they generally behave toward their children, using bipolar items, where poles are anchored with a structured and a permissive stance. This subscale has been associated with observations of laxness (*r* = .61) and shown to identify mothers having difficulties in handling their children. The internal consistency of our French version was good (Cronbach alphas T1/T2 = .75/.72) in our pilot study.

Six items of the Laxness subscale of the *Parenting Scale* [61] were adapted to measure children’s perception of the extent to which their participating parent is permissive or setting limits. The internal consistency was close to satisfactory for this scale (Cronbach alphas T1/T2 = .57/.56) in the pilot study.

Ten items of the Care subscale of the *Parental Bonding Instrument* [62] were translated to measure parental care and involvement (vs. rejection). This instrument has been positively related to an observational measure of parental care [62]. The internal consistency of our French version was good (Cronbach alphas T1/T2 = .79/.77) in the pilot study.

The Care subscale of the *Parental Bonding Instrument* [62] was adapted to gather children’s perception of their participating parent’s care and involvement, contrasted with indifference and rejection, and its internal consistency was also good (Cronbach alphas T1/T2 = .76/.70).

The *Autonomy-Supportive Parenting Skill Scale* was designed within our pilot study [41]. Twelve autonomy-supportive skills taught in the How-to Parenting Program are contrasted with various controlling strategies parents typically use. Parents rated bipolar items, where one pole is anchored with an autonomy-supportive response and the other with a controlling reaction. The internal consistency was acceptable at T1 and good at T2 (Cronbach alphas T1/T2 = .64/.81) in the pilot study.

Parents also completed the *Parental Attitude Scale* [63] to rate their attitude toward AS and psychological control. This scale has predictive validity and has been associated with observational measures of autonomy-supportive and controlling behaviors [63]. In the pilot study, the French version showed good internal consistency (Cronbach alphas T1/T2 = .76/.73).

Children completed an adapted version of the *Perceived Parental Autonomy Support Scale* [31] to assess their perception of the extent to which their parent uses autonomy-supportive and controlling strategies. This scale has a sound factor structure, demonstrates convergent validity, and predicts psychological adjustment. In the pilot study, its internal consistency was good (Cronbach alphas T1/T2 = .70/.78).

##### Parental mental health

Parents were asked to assess their own mental health at each assessment time. Symptoms of anxiety and depression were measured with the *General Health Questionnaire* [64] while negative affect and guilt were measured with the negative affect subscale of the *PANAS* and the guilt subscale of the *PANAS-X* [54, 65]. Parents also assessed positive indicators of their mental health, by rating their positive affect (with the *PANAS* [54]), life satisfaction (with the *Satisfaction with Life Scale* [58, 59]), perceived competence (with the Competence subscale of the *Basic Need Satisfaction in Relationships Scale* [66]) and their self-esteem (with the *Rosenberg’s Self-Esteem Scale* [56]).

#### Potential covariates

At pre-intervention, parents provided demographic questions to determine their age, gender, education level, family income, marital status, first language, ethnicity and number of children. Parents also indicated their children’s age and gender, and evaluated their child’s temperament (*Children’s Behavior Questionnaire* [67]). The measures used in the present study are summarized in an additional table file [see Additional file 1].

### Retention promotion

A 20$ (CAN) compensation was offered to parents each time they completed a questionnaire. After each questionnaire, participating teachers received 10$ (CAN) and children received a gift-certificate of 10$ (CAN) from a popular bookstore. Group facilitators were not compensated for filling out their own short questionnaire but were paid for their work.

### Statistical methods

We will adopt an intent-to-treat approach [68] as this approach increases external and internal validity. Our data will also be hierarchical in nature; pre-intervention, post-intervention, 6-month and 1-year follow up measures will indeed be nested within each parent, who are nested within a parenting group. Accordingly, multivariate hierarchical linear modeling (HLM) analyses will be conducted to test our hypotheses; these analyses have the advantages of estimating error terms while taking into account the nested nature of the data and allow for missing data without decreasing power. Analyses will thus include all participants who completed at least one assessment, regardless of the number of sessions they attended (for the experimental groups).

To evaluate change over time, the four assessment points (pre-intervention, post-intervention, 6-month follow-up, and 1-year follow-up) will be treated as repeated measures to estimate change between each subsequent time points [69]. This approach is chosen because it allows rates of change to differ across time.

#### Preliminary analyses

Although we randomly assigned participants to one of the two conditions, there is still the possibility that some sample characteristics were unequally distributed across the two conditions. We will thus compare the experimental and control groups on baseline variables that may directly or indirectly impact the effect of the intervention (e.g., children’s age and familial SES), to investigate statistical equivalency. If an important imbalance is found between our conditions, we will control for this/these variable/s in later analyses. We will also investigate the percentage of variables not equally distributed between our two conditions and then judge the success of our randomization.

#### Primary analyses

The effect of the How-to Parenting Program will be evidenced by significant interactions between rates of change and experimental conditions. These interactions should reveal that child mental health increases over time for participants in the experimental condition, but not for participants in the control condition. Based on our pilot study, we expect that the program will be effective to decrease parent-reported I and E problems.

#### Secondary analyses

Also based on our pilot study, we expect that the program will help reduce teacher-reported I and E problems, and that it will foster higher strengths and well-being [41, 43]. We will also test the impact of the program on parenting and parents’ mental health as well as conduct mediation analyses of the putative impact of the intervention. We expect that improved parenting, as a consequence of the program, will lead to improved child mental health. Finally, we will conduct moderation analyses to explore whether the program’s impact varies according to certain characteristics of children (e.g., age, gender), parents (e.g., age, SES), groups (e.g., content fidelity) or circumstances (e.g., whether one or two parents took part in the program; whether another child and/or family intervention was received).

#### Handling missing data

An important advantage of HLM is the use of estimation procedures that allow for missing data without decreasing power (e.g., full information maximum likelihood estimates). All participants with at least one assessment will thus be included in our analyses because their missing data is estimated from the information of other participants. This procedure has been shown to yield unbiased coefficients, whether data is missing at random or completely at random [70]. Nevertheless, participants with and without missing data will be compared to document the pattern of missing data. Though we planned a variety of procedures to maximise the retention rate and increase the exactitude of our estimates (e.g., compensation, phone contacts), identifying the characteristics of participants who tend to dropout is useful to adjust future retention strategies.

## Discussion

In this study, we aimed to improve child mental health via a parenting group delivered in school settings. The “How to talk so kids will listen & listen so kids will talk” program [36] was chosen because it addresses all of the three main dimensions of optimal parenting (i.e., structure, affiliation and AS) in seven structured sessions. We aimed to determine whether the How-to Parenting Program optimises child mental health during middle childhood, compared to a wait-list condition.

Because solely assessing psychological problems provides an incomplete view of mental health, we assessed how symptoms and positive indicators of mental health changed over time. Children’s social skills and well-being are rarely measured in parenting program studies and when they are, effects are smaller than for psychological problems [32]. Since the How-to Parenting Program targets parenting rather than child misbehavior and since it integrates AS in addition to structure and affiliation, we expect that it will lead to improvements in children’s socio-emotional strengths and subjective well-being, in addition to decreases in their I and E problems. The latter two types of problems are expected to be equally impacted by the program, since effects obtained in our pilot study were of comparable size [41, 43]. We expect various aspects of children’s mental health to be positively influenced because the program doesn’t target one aspect of children’s functioning (e.g., obedience) at the expense of another (e.g., well-being).

Many programs do not integrate the AS dimension of optimal parenting. This may be due to the fact that most of them were first designed to treat disruptive E problems, before adaptations for the general population were made. Indeed, embedded in a behavioral psychology perspective, the vast majority of parenting programs target structure and affiliation [15, 33–35] but not AS. These programs focus on contingent parental attention in order to decrease undesirable child behaviors (e.g., planned ignoring) and increase desirable ones (e.g., positive reinforcement) and focus on directed play, coaching, praise and incentives to foster better parent-child relationships and more consistent and less coercive discipline [71, 72].

Another reason that could explain the relative absence of AS is the unfortunate imbalance among parenting dimensions in basic parenting research. Indeed, AS has received less attention than its opposite, psychological control [73–75]. To document the role of AS in fostering human development and health, we integrated knowledge from decades of fundamental and applied research conducted within Self-determination theory [76–78]. In light of compelling evidence for the benefits of AS, we selected a program that addresses this fundamental dimension, along with structure and affiliation.

### Evaluated vs. original how-to parenting program

Our pilot study [41] and the present RCT used the French version of the How-to Parenting program. Although the French version is highly similar to the original English program, slight differences between the two versions will nevertheless prevent direct generalizability of our efficacy results. Specifically, the French workshop consists of the written translation of the audio (CDs) English material. Since everything is said by facilitators, the translated workbook for leaders [44] is much longer than the English one. We thus decided to rely on teams of two trained co-facilitators. Training facilitators is another difference between the present implementation and the original one. The program’s authors originally presented their material using audio or video recordings such that the facilitators did not require any specific training. For the translated version, the authors still do not recommend training because group leaders have access to the verbatim of the recordings and this manualized material is presented in a highly straightforward and accessible way. The reason we provided training was to promote adherence.

Another major difference between the program format evaluated in this RCT and the original English workshop format is the reliance on only one book. Indeed, the original workshop format involves reading weekly extracts from another book, “Liberated Parent, Liberated Children: Your Guide to a Happier Family” [79, 80] each week, in addition to reading extracts from the main “How-to” book. The present RCT did not use the additional book, to shorten required readings in the hope of fostering parents’ homework completion and their sense of competence. In our former pilot study using both books [41], participants rarely completed all of the assigned readings. The second book was thus simply recommended as an enriching resource for the RCT. Given these slight differences, a replication RCT will be needed to ensure that our results can generalize to the English version.

### Present RCT vs. pilot study

Results from our pilot study were promising and we expect similar effects in this study. However, if results were to differ, methodological differences between these two studies should be considered. First, as mentioned, the pilot study relied on two recommenced books whereas the present RCT relied only on the one describing the workshop. Second, the pilot study had a pre-post intervention design whereas the present study is a RCT. Third, there were eight sessions in the pilot study instead of the seven sessions in the RCT (in the pilot study, there was no information meeting and T1 questionnaires were completed during an additional introductory group session). Fourth, whereas the pilot study only recruited parents and children, the present RCT also invited children’s teachers to assess children’s behaviors. Fifth, whereas children only filled out questionnaires at pre- and post-interventions in the pilot study, they were also invited to do so at 6-month and 1-year follow-ups in this RCT, just as parents and teachers. Sixth, in the pilot study, parents who had more than one child at the participating school chose which child was the “targeted” child (i.e., “the child you were thinking of when signing up for the program”) whereas this choice could not be made in the RCT. Seventh, whereas parents of the pilot study completed their T2 questionnaire during their last session, parents participating in the RCT were invited to complete their T2 questionnaire at home. Finally, whereas there was no compensation in the pilot study, participants of the RCT were compensated for their time filling out questionnaires.

### Strengths

In addition to assessing an intervention that taps the three key aspects of optimal parenting, the present study has other important strengths. The study uses multiple informants, along with a large sample size and longitudinal follow-ups, even though for teacher-reports, respondents will have changed within the year-long study. Rigorous methodological procedures also include the distinction made between the implementation and the evaluation team members in order to limit social desirability, the blindness of teachers and RAs, and the fidelity assessments. Other ethical and methodological strengths include the non-stigmatizing invitation to all parents, the absence of exclusion criteria based on parents’ or children’s difficulties and the practical choice of local schools for the study’s setting, instead of clinics. Choosing to recruit and offer the program in local schools also helped to make this intervention non-stigmatizing. In a related vein, given that this study was designed as a universal rather than a selected preventive intervention, parents who had more than one child in grade school could not decide which child would be evaluated. This procedure avoided potential sampling biases, such as having a higher proportion of higher-risk children in the sample.

Importantly, the study has high ecological validity, as it was offered by a heterogeneous group of facilitators, with some who, apart from receiving a 3-day training, had no extensive background in program implementation and/or optimal parenting. Since this minimal training was an exposure to the program’s content, former participating parents could easily become facilitators, a relatively simple and low-cost way to foster sustainable dissemination. Together, the relatively low cost of the manualized material, the minimal training required (compared to extensive training and/or expensive certifications) and the possibility of offering the program within schools greatly increase the potential outreach of this program, thereby facilitating accessibility of parenting resources to interested parents. In general, though the present study is not a cost-effectiveness project, the large effect-sizes reported in our pilot study conducted with a population-based sample supports that it may be worth using this program as a universal approach to preventing child psychological problems and improve child mental health by fostering optimal parenting.

### Limits foreseen

The main limitation of the present study is the absence of observational measures. Though a multi-informant approach was used, it would have been optimal to use observations of parents and their children in their home and school environments, made by independent coders. Given that not all parents are comfortable with having their interactions with their children observed, such measures were not included to encourage participation and thus increase the external validity of our findings.

Another potential limitation is the relatively small number of children old enough to provide information about the parenting they receive and their own well-being. Including child-reports was of utmost importance for us, since children are better positioned to describe their emotions, self-esteem, life satisfaction and the parenting they receive. However, the minimum age required to fill out questionnaires with high validity (i.e., 8 years old) diminishes the number of child-reports and the statistical power to detect effects on children’s perceptions and experiences.

Third, a key challenge encountered by our study team was the conflicting recruitment and differentiation goals. In each school, information meetings were decisive and there was a fine line between giving parents a taste for taking part in the study, without eliciting undue disappointment among parents who would later be allocated to the wait-list. Perhaps that after learning about the existence of the How-to Parenting Program, though it was presented in highly general terms, some parents were motivated to learn the skills and decided to buy the book, without waiting for their workshop, thereby increasing the risk for contamination or poor condition differentiation. Indeed, some parents asked principal investigators if they were “allowed” to buy the book. When this happened, we answered that no research can dictate what participants can/should do, and that questions about their choice on this matter would be included in the questionnaire.

A last methodological concern pertains to potential ceiling effects. Since parents of more than one child could not choose which child would be the targeted one, they could not rate the behaviors of the child they were perhaps thinking of when signing up for the study. There is a lower probability of detecting improvements in families in which the targeted child was the one with the better mental health.

## Conclusion

Results of this study will provide initial evidence about the efficacy of the French version of the How-to Parenting Program, originally created in the Unites States. The present RCT will evaluate its impact on children’s E and I problems as measured by the CBCL which will allow informative comparisons with other evaluated parenting programs. If proved efficacious, the study will represent an important first step in suggesting the How-to Parenting Program as a promising way to foster children’s mental health. We believe the results of this study will ultimately inform the way we can improve children’s mental health and well-being, by helping parents of the general population provide more skillful structure, higher affiliation and importantly, more AS.

## Additional file

Additional file 1: Summary of instruments used in the How-to Parenting Program RCT. Measures used in the RCT are summarized (XLSX 13 kb)

## Abbreviations

AS: Autonomy support
CAN: Canadian
CBCL: Child Behavior Checklist
CD: audio format
CR: Child report
DVD: video format
E: Externalizing
FU: Follow-up
HLM: Hierarchical linear modeling
I: Internalizing
PANAS: Positive and Negative Affect Schedule
PR: Parent report
Q: Questionnaire
RA: Research assistant
RCT: Randomized controlled trial
SES: Socio-economic status
T1: pre-intervention
T2: post-intervention
T3: 6-month follow-up
T4: 1-year follow-up
TCRS: Teacher-Child Rating Scale
TR: Teacher report
